# Occupational Attainment Among Parents in Germany and the US 2000–2016: The Role of Gender and Immigration Status

**DOI:** 10.1007/s11113-022-09744-0

**Published:** 2022-10-27

**Authors:** Paige N. Park

**Affiliations:** Department of Demography, University of California, Berkeley, CA USA

**Keywords:** Gender, Immigrant, Occupational status, Occupational inequality, Policy

## Abstract

In many OECD countries, women are underrepresented in high status, high paying occupations and overrepresented in lower status work. One reason for this inequity is the “motherhood penalty,” where women with children face more roadblocks in hiring and promotions than women without children or men with children. This research focuses on divergent occupational outcomes between men and women with children and analyzes whether parental gender gaps in occupational status are more extreme for immigrant populations. Using data from the Luxembourg Cross-National Data Center, I compare changes in gendered occupational segregation from 2000 to 2016 in Germany and the USA among immigrant and native-born parents. Multinomial logistic regression models and predicted probabilities show that despite instituting policies intended to reduce parental gender inequality in the workforce, Germany fares worse than the USA in gendered occupational outcomes overall. While the gap between mothers’ and fathers’ probabilities of employment in high status jobs is shrinking over time in Germany, particularly for immigrant mothers, Germany’s gender gaps in professional occupations are consistently larger than gaps in the US. Likewise, gender gaps in elementary/labor work participation are also larger in Germany, with immigrant mothers having a much higher likelihood of working in labor/elementary occupations than any other group—including US immigrant women. These findings suggest that work-family policies—at least those implemented in Germany—are not cure-all solutions for entrenched gender inequality. Results also demonstrate the importance of considering the interaction between gender and other demographic characteristics—like immigrant status—when determining the potential effectiveness of proposed work-family policies.

## Introduction

In the Global North, women are more educated, politically active, and economically participative than ever before (World Economic Forum, 2020), but despite these marked improvements, persistent patterns of inequality remain (World Economic Forum, 2020). Gender gaps in occupational status still exist in many countries around the world, which is problematic since occupational prestige is closely tied to career and material stability, class identity, and wages (Conley & Yeung, 2005; Gangl & Ziefle, 2009). Mothers in particular face additional barriers to entering into and progressing within certain occupations, a concept scholars deem the “motherhood penalty” in the literature (Budig et al., 2012; Correll et al., 2007). To combat these persistent divides, many OECD countries have implemented policies at least partially intended to eradicate gender gaps in the economic sphere, offering paid parental leave and public childcare options for young parents (OECD, 2021a). Although there is substantial evidence supporting the hypothesis that some policy formulations are beneficial for mothers, other research shows that government-led solutions are not universally effective and have sometimes exacerbated disparities between low-and high-SES women (Mandel, 2011; McKay et al., 2016; Misra et al., 2011).

Research on the heterogeneous effects of family work policies on population subgroups tends to focus on income-related disadvantages over other types of vulnerability. This study expands the literature on subgroups by examining immigrant parents’ occupational status following family work policy changes. Immigrant mothers may face unique barriers to entering and advancing in employment that current policies may not account for, including lack of social networks, gender norms in native countries, and cultural and legal barriers that make career advancement more difficult (Abrego & Lakhani, 2015; Gomberg-Munoz, 2017; Hagan, 1998; Villares-Varela, 2018). Understanding how immigrant mothers experience additional penalties in the workforce and how they respond to policy changes is a crucial next step in the unfolding conversation around gendered occupational segregation and family work policy.

While there is considerable research on motherhood penalties and immigrant occupational outcomes there is little research that combines these topics and studies them over time and cross-nationally. Research shows that mothers and immigrant women face barriers to entering high status positions at work (Djamba & Kimuna, 2012; Jee et al., 2019; Villares-Varela, 2018), but few studies focus on barriers faced by women who are immigrants *and* mothers *and* employed in the paid labor force. The large size of the LIS data allows for multiple layers of subgroups analysis like this, while other datasets may lack this kind of robustness. The rich data used in this study allows for greater understanding of the heterogeneity within the larger group of employed individuals in these countries. Repeated cross sectional (or longitudinal) and cross-national studies measuring occupational status among parents, especially parental subgroups like immigrant parents, are sorely needed as work-family policies continue to evolve in OECD countries. Thus, this paper’s contribution centers around the rich contextual information it provides about parental subgroups that can improve conversations among policymakers and academics about status inequities in the Global North.

Using repeated cross-sectional data from the LIS Cross-National Data Center’s Luxembourg Income Study (LIS), I examine gender and immigrant inequality among parents in the labor force, by investigating gender/immigrant gaps in occupational status in two OECD countries: Germany, a country that *has* implemented several governmental policies to address gender inequality, and the US, a country that *has not* implemented similar federal policies. More specifically, the three research questions I consider are:

1. How does the probability of parents’ employment in high/low prestige occupational positions differ by gender and by immigrant status in two countries with vastly different family work policy contexts?
2. How does the relationship between occupational status and gender/immigrant status change over time?
3. Does the identity of “mother” interact with the identity of “immigrant” to predict lower occupational prestige?

The comparison of occupational status in this study focuses on four primary variables: parent gender (mothers compared to fathers), parent immigrant status (foreign-born compared to native-born), country/policy context (Germany compared to the USA), and time (2000–2016). I find that immigrant status and gender both matter for predicting occupational status and that they tend to matter more together. Immigrant mothers are the most disadvantaged in terms of occupational status in both countries. However, in the USA, immigrant fathers approach the same level of disadvantage by 2010 and 2016, and in Germany, immigrant mothers are more likely to be in professional work, both absolutely and relative to native-born mothers. Despite improvements, gaps in gendered-immigrant occupational status are larger in Germany than in the USA at all time points, despite the policies they have implemented to counteract these trends.

I begin the paper with an overview of the literature on female and immigrant disadvantages in the labor force, particularly disadvantages related to motherhood. Then, I describe the public policy solutions many countries have implemented to ameliorate some of these career impediments. Next, I delve into the primary research questions of the study that I briefly presented above. I then describe the similarities and differences in the economic, social, immigrant, and policy landscapes in Germany and the USA to allow for better interpretation of what might have led to divergent results in the two countries. Finally, the methods, results, and discussion and conclusions sections outline my process of analysis, the findings of the study, and the contribution of these results to academic and policy conversations.

## Gendered Occupational Segregation and Motherhood Penalties

Social scientists and feminist theorists largely agree that gender continues to be a mechanism of social stratification in many OECD countries. Some theorists argue that although more women are entering the workforce than ever before, they are entering on “male terms,” and are subject to the hegemonic masculine nature of workplaces and bureaucracies (Connell & Messerschmidt, 2005; Hochschild, 1997). In other words, the workforce, which primarily employed men with stay-at-home partners for much of the modern age has not fully adjusted to increases in mothers’ employment. As a result, social and structural pressures on women to leave the workforce, or at least reduce their working hours, are strong, as women continue to take responsibility for the bulk of childrearing and household duties (England, 2010; Hochschild, 1989; Pailhé et al., 2021), face discrimination in promotion decisions (Ibarra et al., 2010; Javdani & McGee, 2019), and make less on average than their male counterparts (Blau & Kahn, 2017; Christofides et al., 2013).

Surprisingly, rather than “opting out” of the male-centric workforce for its failure to accommodate their needs (Percheski, 2008), women often stay, instead gravitating towards traditionally female occupations, or feminine fields within traditionally male occupations—like social science or humanities in academia—with only some of the most highly educated women entering traditionally male-dominated occupations (England, 2010). “Female occupations” often include those that focus on working conditions rather than pay, like public sector jobs, which tend to focus on benefits like flexible hours and long parental leaves. Women tend to gravitate towards careers and positions that are more “family-friendly,” while men are more comfortable with careers that require long and rigid working hours, and where there are usually financial rewards that coincide with putting in long hours (Goldin, 2014; Kleven et al., 2019a, 2019b).

Women’s gravitation toward family friendly careers may be largely explained by “the particular [way] in which getting a living is integrated with raising children” (Elson, 1995; 7). Because biology often forces women with children to take primary responsibility for a new baby (through pregnancy, birth, and breastfeeding), Elson (1995) theorizes that they are more likely to get locked into the later phases of childrearing as well. This theory is supported by empirical evidence showing that even after the “gender revolution” associated with women’s dramatic movement into the paid workforce, mothers often continue to take on the bulk of unpaid work in the home (Pailhé et al., 2021), and the most egalitarian households are often those in which fathers became involved in childrearing during the earliest stages of a child’s life (Earle & Heymann, 2019; Patnaik, 2019; Raub et al., 2018).

The expectation that mothers will shoulder most of the caregiving responsibilities leads employers to perceive mothers as less reliable and they may hesitate to hire or promote women with children (Correll et al., 2007). The difficulties women face in gaining, maintaining, and progressing in their careers after having children contribute to a concept known as the “motherhood penalty,” where mothers face systematic disadvantages in hiring, pay, and promotions, among other things (Budig et al., 2012; Correll et al., 2007; Jee et al., 2019). In contrast, fathers do not tend to face the same penalty after having children and are often largely unaffected professionally by a new birth (Kleven et al., 2019a, 2019b). The female penalties associated with having children explain much of the remaining gender gaps in earnings and are closely associated with gaps in other indicators like occupational status and promotion (Kleven et al., 2019a, 2019b). Additionally, the contribution of child penalties to workplace gender inequality has increased dramatically since the 1980s (Kleven et al., 2019a, 2019b). Figuring out how to lessen the impacts of child penalties on women’s professional lives is essential for workplace equality and for sustainable family life.

## Immigrant Mothers and Gender Inequality

Workplace gender bias and motherhood penalties may be even more severe among immigrant populations in various OECD countries. While both male and female immigrants, even highly educated immigrants, struggle to have job mobility and attain occupational prestige (Fellini et al., 2018; Golash-Boza, 2015; Hall et al., 2019; Kanas & van Tubergen, 2009), immigrant women face unique barriers that further marginalize them. They are more likely than immigrant men to limit their career goals in favor of their spouses’ goals, to lack social capital, and to decide to stay home with children because of strict gender norms in origin countries (Gomberg-Munoz, 2017; Hagan, 1998; Villares-Varela, 2018). As a result, employment rates and earnings tend to be lower among immigrant women (Blau et al., 2011; Browne & Misra, 2003; Djamba & Kimuna, 2012; Man, 2004; Sprengholz et al., 2021), despite the increasing social legitimacy of female labor migration (Oishi, 2005).

Building on the immigrant labor market literature, this research investigates gender occupational inequality among immigrant parents in the USA and Germany. Intersectional research on gender occupational segregation in the US often examines racial and ethnic differences rather than immigration status, or it isolates gender-immigrant studies to single racial groups like Hispanic or Black migrants (Mintz & Krymkowski, 2010; Pettit & Hook, 2009; Tesfai & Thomas, 2020). These studies predictably find that having multiple marginalized identities (e.g. being Black and an immigrant and a woman) creates more barriers to occupational equality than only having one marginalized identity (Tesfai & Thomas, 2020). In Germany, many longitudinal studies on immigrant occupational outcomes consider *citizenship status* rather than immigrant status (Pettit & Hook, 2009; Sprengholz et al., 2021; Winkler, 2019), which is a critical limitation in German immigration research. Of the gender research in Germany that does consider immigrant status explicitly, few studies include cross-national research components (Fleischmann & Höhne, 2013). The current study addresses this limitation in the literature by comparing *immigrant* occupational outcomes by gender in Germany and the US over time. This research also investigates mothers’ and fathers’ outcomes exclusively and references work-family policies as a potential factor influencing occupational outcomes, another element not included in many other studies on gender-immigrant occupational status disparities.

In sum, while previous research has analyzed gender (and motherhood) gaps in occupational status cross-nationally and over time (Abendroth et al., 2014; Budig et al., 2012), immigrant gaps in occupational status cross-nationally and over time (Chiswick et al., 2003; Pichler, 2011; van Tubergen, 2006; Winkler, 2019), and immigrant status and gender, few studies have analyzed all four of these elements (gender and motherhood, immigrant status, country context, and time) *simultaneously*. This research will expand the literature by including these four components and by limiting the analysis to employed parents to better capture possible work-family policy influences. By investigating the disparities that exist not only between mothers and fathers, but also among native-born parents and immigrant parents, this study can better inform family work policy conversations.

## Public Policy Solutions

Because motherhood plays such a significant role in female employment outcomes, policymakers have often focused reform efforts on family friendly work policies, like parental leave and government-funded childcare. The least successful policies are those that offer mothers too much time off after a baby is born because the longer mothers spend out of the workforce, the more likely they are to stay at home long term, to take part-time positions, and experience downward movement in their careers (Aisenbrey et al., 2009; Baker & Milligan, 2008; Blau & Kahn, 2013; Pettit & Hook, 2009). Moderate length leaves, by contrast, may have positive effects on women’s career outcomes (Misra et al., 2011).

Family leave policies can also harm women when they are strictly reserved for mothers, by sending the message that family responsibilities are a woman’s affair and that only mothers should expect to take time off when a baby is born (Adema, 2013). In recent years, many countries have taken steps to counter this idea by establishing policies that incentivize fathers to take parental leave, using tactics like “daddy quotas,” where fathers must either take a portion of the leave or couples lose the benefit altogether (Dunatchik and Ozcan, 2020; Patnaik, 2019). Some research shows that father quotas are quite effective at reducing long term gender inequality in the workforce—and within heterosexual relationships at home—since fathers are much more likely to take the leave when it is reserved for them (Budig et al., 2016; Dunatchik and Ozcan, 2020; Mayer & Le Bourdais, 2019; Patnaik, 2019; Raub et al., 2018).

Public childcare policies may also be more effective at improving female labor market outcomes, like occupational status, because public childcare is a work-facilitating policy, rather than a work-reducing policy (Budig et al., 2016; Pettit & Hook, 2009). In other words, providing a free childcare service for working mothers, as opposed to offering long parental leaves where mothers can stay home with children, allows women to return to work faster after having a child. Thus, the most successful work-family policies typically offer shorter leaves to mothers, encourage fathers’ involvement in parenting, and support mothers as they return to work.

Yet, other scholars argue that even the more successful policies may fail to remove gender barriers in some contexts. For example, in Austria, where officials have implemented various policies including parental leave and public childcare, the policies have had “virtually no impact on gender convergence” in labor market outcomes (Kleven et al., 2020, p. 1). Research also finds that vulnerable groups, like lower-SES families, may not take advantage of or benefit from work-family policies as much as higher-SES families do (Geisler & Kreyenfeld, 2019; McKay et al., 2016; Misra et al., 2011). Likewise, while public childcare is one of the best ways to facilitate mothers’ returns to work, this is mostly true of women with at least a vocational degree (Zoch, 2020). The provision of free childcare is less effective for low-income and less educated women (particularly immigrant women) whose non-standard schedules make it difficult to find available childcare during their working hours (Sandstrom & Chaudry, 2012). Immigrant fathers are also less likely than native-born fathers to take parental leave when it is offered (Ellingsæter et al., 2020; Tervola et al., 2017) and immigrant mothers may hesitate to take up family leave policies when they are intertwined with immigration policies (Straut‐Eppsteiner, 2021). These studies suggest that many work-family policy formulations may not improve occupational outcomes across the board, especially for lower income, less educated, and foreign-born individuals. Determining when and why policies are not effectively closing gender gaps is necessary for advancing gender equality in the labor force.

## Research Questions

While I cannot directly answer the question of how policies *caused* different outcomes in gender-immigrant occupation patterns, I can address how these patterns differ between two similar OECD countries with quite different approaches to work-family policy. I will compare occupational outcomes from 2000 to 2016 in Germany, with its more extensive and far-reaching work-family policies, and the USA, with its less centralized and more haphazard approach. With that in mind, this study investigates (1) how gender-immigrant parental occupational status might differ in two countries with vastly different policy contexts, (2) how immigrant status and gender influence the occupational status of parents over time, and (3) how immigrant status and gender interact with each other to predict occupational status. Work-family policy research suggests that in countries with more generous and work-facilitating policies and where support for the policies is high, particularly in countries with “daddy quotas” and public childcare, women may have more opportunities for career advancement and fewer barriers restricting them from pursuing more prestigious full-time careers (Budig et al., 2012; Dunatchik & Özcan, 2020). Research also suggests that work-family policies are generally less effective for disadvantaged populations like migrants (Geisler & Kreyenfeld, 2019; McKay et al., 2016; Misra et al., 2011; Sandstrom & Chaudry, 2012).

Surprisingly, I found that gaps in occupational status between mothers and fathers were *larger* in Germany than gaps in the US across time, suggesting that even in a context with more macro-level policies in place, disparities in occupational status still exist. I also found that immigrant mothers were often over-represented in the lowest status jobs and under-represented in the highest status jobs in both countries regardless of policy context. This finding indicates that the immigrant experience should be considered in workplace gender equality conversations and policy development.

## Study Context: Germany and the USA

Germany and the USA are ideal contexts to investigate my research questions because while the countries have relatively similar economic systems, sociocultural norms, and migrant-dense populations (CIA, 2021a, CIA, 2021b; Fuwa, 2004; Wilde & Diekman, 2005), they have very different work-family policy approaches. I detail the economic situation, social and gender norms, immigration history, and public policy approach of each country below.

### Economy

The economic systems in Germany and the US are similar in terms of employment rates and are two of the largest, strongest, and most influential in the world, making them excellent choices for international comparisons. Over the last 20 years, employment rates—among the working-age population (defined by the OECD as 15 to 64)—in the USA and Germany ranged between 65 and 77%, though the US employment rate has decreased over the period from 2000 to 2020—from 74 to 67%—while the employment rate in Germany has increased—from 65 to 77% (OECD 2021b). The US and Germany both have large economies in terms of GDP (CIA, 2021a; CIA, 2021b). While the US economy is much larger than the German economy, they both fall within the top four largest economies in the world (IMF, 2021). Out of these four economies (China, US, Japan, Germany), the US and Germany are the most similar in their major industries, government systems, *and* cultural and social norms, making them the best comparison groups among these large economy countries.

In terms of dominant industries, the two countries are more similar to each other than they are to countries with emerging markets, but different enough to require consideration in the context of this paper (CIA, 2021a; CIA, 2021b). Germany’s industry sector (economic activities that produce material goods) is larger than the industry sector in the US with 24% of the German population working industry jobs compared to 19% of the American population (CIA, 2021a; CIA, 2021b). By contrast, the service sector (economic activities that don’t produce material goods) in the US is larger than the service sector in Germany with 80% of the American population working in the service sector compared to 74% in Germany (CIA, 2021a; CIA, 2021b). This difference between industries reflects Germany’s greater emphasis on export-focused trade, and their resulting trade surplus. The US, on the other hand, has a trade deficit. Around 1% of the population in both countries is employed in the agricultural sector (CIA, 2021a; CIA, 2021b).

Another important difference between the German and the US economies is their response to and recovery from the Great Recession, the most salient exogenous shock that occurred during the time frame of this study. While the recession affected both countries, the impacts on the US were much longer lasting and more severe. Though the time frame of the recession is usually cited as falling between 2007 and 2009, the US recovery was slow, with real GDP not recovering to pre-recession levels until 2011 and employment rates not recovering until 2014 (FRED, 2021a, FRED, 2021b). Experts attribute the severity of the recession to major housing sector damage, credits for borrowing and spending not being as readily available, and government spending not being adequate enough to offset losses in the private sector (Bernanke, 2012). Germany on the other hand emerged from the recession incredibly strong, without experiencing an employment decline or an increase in unemployment (Rinne & Zimmermann, 2012). In fact, by 2009, Germany’s labor market became stable, and GDP began to steadily grow from 2010 onwards (Rinne & Zimmermann, 2012).

### Social and Gender Norms

Another similarity between the US and Germany, at least relative to many other countries around the globe, are their social and gender norms. American and German perceptions of men and women, as well as their slow shifts towards more egalitarian gender role attitudes, closely resemble each other (Lee et al., 2007; Scott & Braun, 2009; Wilde & Diekman, 2005).

However, the similarities that justify the use of Germany and the US as comparison groups cannot completely account for smaller differences in gender attitudes and gendered trajectories in the two countries. Work-family trajectories are highly gendered in Germany and are less gendered in the US, especially when it comes to the highest prestige occupations. That is, because of family related barriers, German women may not be as likely as American women to be in high-status jobs (Aisenbrey et al., 2009). Women in Germany might also be less committed to their careers than women in the US, leading them to opt-out of the labor force more often, as is the case during the unfolding COVID-19 pandemic (Gangl & Ziefle, 2015; Reichelt et al., 2021). In these ways, gender roles in Germany may be more rigid than gender roles in the US. On the other hand, the annual Global Gender Gap Report ranks Germany higher in gender equality than the US (Germany is number 10 while the US is number 53) (World Economic Forum, 2020). The World Economic Forum explains that Germany’s high ranking is largely due to its political gender equality and that the US’s poor ranking is a result of progress towards gender parity stalling (see also: Scott & Braun, 2009). The large wage gap and the lack of women in the top business positions also contribute to the US ranking. Thus, it is unclear which country “wins out” in terms of gender equality; they both have strengths and weaknesses.

One final difference between the two countries’ gender and work norms is their utilization of the family network for childcare. In the US, parents are slightly more likely than parents in Germany to employ informal childcare options, like the family network, especially when their kids are two or younger (Laub, 2016; OECD, 2021d). This difference makes sense in the context of policy differences between the two countries; there are free formal childcare options in Germany, but not in the US (SPLASH, 2014).

### Immigration

Germany and the US are both home to extremely large numbers of immigrants; nearly 45 million immigrants reside in the US, which is equivalent to about 14% of the US population, and around 13 million immigrants live in Germany, about 20% of their population (Batalova et al., 2021; United Nations, 2019). They both receive high influxes of immigrants each year as the top two migrant-receiving countries in the world (United Nations, 2019), and as such, the migrant stock in both countries has increased considerably over the time period of the current study (Budiman, 2020; Statista, 2021). While both are popular immigrant destinations, the demographic characteristics of immigrants to the US and Germany are quite distinct. The composition of US/German migrants have varied over the years, but immigrants to Germany are most commonly white Europeans, coming from Eastern European countries like Turkey, Poland, and Romania (Destatis, 2019), while immigrants to the US are more diverse in terms of race and ethnicity, and are dominantly Hispanic and Asian, primarily from Mexico, China, India, and the Philippines (Budiman, 2020). Additionally, women make up a slight majority (around 51%) of the foreign population in both Germany and the US (OECD, 2021c).

Since immigrant groups in Germany and the US are coming from such different areas of the world, considering differences in the social or gender norms of native countries is necessary. For example, on the World Economic Forum’s ranking of gender equality (where 0 is no parity and 1 is total parity), Eastern Europe scores 0.732 on “Economic Participation and Opportunity” while Latin America scores 0.642 and South Asia scores a mere 0.365 (World Economic Forum, 2020). It is possible then that immigrants to the US may have internalized stricter gender norms around work than immigrants to Germany, which may affect immigrant women’s occupational outcomes.

Though immigrants to the US and Germany may be demographically and culturally distinct, they often take similar occupational roles in both countries. Immigrants generally struggle to completely integrate into the labor market, and are less likely to be employed and more likely to take lower status positions than native-born residents in both Germany and the US, even after controlling for education (Eckstein & Peri, 2018; Heilbrunn et al., 2010; Kogan, 2011; Winkler, 2019). Occupational fields with particularly large shares of immigrants—particularly Hispanic migrants in the US and African and Turkish migrants in Germany—include agriculture, construction, manufacturing, and unskilled jobs (Eckstein & Peri, 2018; Kogan, 2011), though Chinese and Indian migrants in the US are most often employed in high-skilled occupations like computer programming and managerial work (Eckstein & Peri, 2018). Influxes of highly educated immigrants to Germany over the past few years contributed to higher occupational attainment among recently arrived cohorts, but occupational status tends to decrease as cohorts are tracked across time, probably due to outmigration of the most skilled migrants (Sprengholz et al., 2021). This suggests that the long-term “stayers” in Germany may have lower occupational statuses than those who leave after a few years (Sprengholz et al., 2021).

Finally, the immigration and citizenship policies in Germany and the US likely play a role in shaping immigrant occupational status as well. In some ways, the two countries have similar immigration laws. Both have instituted measures designed to control “irregular immigration” including penalties for employers of undocumented migrants and fines or deportation for irregular migrants (United Nations, 2017). They have also both regularized legal status under certain conditions to encourage legal migration (United Nations, 2017).). However, the rationale for each country’s policies is distinct and might reflect what types of work each country hopes immigrants will consider. In both countries, immigration policy is motivated by demand for workers in certain sectors of the economy, but for Germany, population decline and population aging are other motivating factors that may funnel migrant workers into geriatric care roles or other jobs related to elder support (United Nations, 2017). In terms of citizenship, the US has unrestricted, *jus soli* citizenship laws whereas Germany’s birthright laws *are* restricted; for a child to gain German citizenship at birth, one parent must have lived in Germany for at least 8 years (United States Office of Personnel Management, 2001). Citizenship is an important aspect of immigrant integration that can shape immigrants’ professional decision-making. Differences in citizenship policy must consequently be considered in the study of migrant occupational status.

### Work-Family and Gender Equality Policies

#### Germany

Within the last 15 years, Germany has instituted three policies expected to greatly reduce gender gaps in employment outcomes. First, in 2007, Germany updated their parental leave policy to include a paternity quota or “daddy quota,” which reserves two months of non-transferable leave for fathers after a child is born. The 2007 policy also specified that parents should receive earnings-related parental leave benefits rather than the mean-tested flat rate benefits that parents received before, meaning that those taking leave would now receive income-dependent payments. In this case, parents would receive 67% of their average earnings—from the year before the child was born—for the months they took off from work (OECD, 2020). All benefits would reset with the birth of each new child (OECD, 2020). Shortly after the policy changes, Germany saw a marked rise in the percentages of fathers who took the leave, indicating that father’s quotas may be more effective than the gender-neutral leave policies of the decades before (Giesler and Krayenfeld, 2019). The 2007 policy also decreased the duration of mothers’ time out of the workforce and increased their overall employment rates and working hours (Spiess & Wrohlich, 2008; Ziefle & Gangl, 2014).

Second, Germany implemented a reform in 2013 that gave all children ages one through three a right to childcare (SPLASH, 2014). This essentially means that affordable or free childcare must be available to every parent in Germany; if the government does not provide childcare, parents have the right to sue (SPLASH, 2014). Public childcare in Germany is provided by non-profit organizations, churches, and city governments—and family daycare centers are typically state-subsidized (SPLASH, 2014). While Germany’s childcare system has always been one of the most affordable of the OECD countries (Immervoll & Barber, 2006), the 2013 policy, in theory, prevents any parents who may have previously lacked access to free or affordable care from having to go without it.

Finally, in 2015, Germany introduced an Act that requires companies to ensure that at least 30% of their 50/50 co-determined supervisory boards (i.e. boards where half of the members are employees) are women (Binder & Zeppenfeld, 2015). If the 30% quota is not met in board elections, the election will be deemed void, and empty seats will remain until the next election (Binder & Zeppenfeld, 2015). Additionally, for a larger group of German companies, the Act requires that they set their own goals for gender composition of the supervisory and managing boards and other leadership positions (Binder & Zeppenfeld, 2015). The only requirements for company-determined goals are that target proportions for women’s participation must not fall below the status quo if less than 30% of current leaders and board members are women, or that women’s participation must not be less than 30% if the status quo is above 30% (Binder & Zeppenfeld, 2015). Targets (and whether or not targets were met) must be published in management reports to keep companies accountable (Binder & Zeppenfeld, 2015).

#### USA

While Germany has implemented far-reaching and evidence-supported work-family policies, the USA has not, or at least not to the same extent. As a result, federal-level policies aimed at helping working mothers (and fathers) have not changed much since the early 90 s. Prior to 2020, the USA had only instituted one federal leave policy: The Family and Medical Leave Act (FMLA). This law, passed in 1993, offers eligible employees 12 weeks of *unpaid* leave after the birth of a child (U.S. Department of Labor n.d). The policy is limited because (1) it doesn’t provide compensation for those who take the leave and (2) it is not universally applicable; only slightly over half of the employees in the US meet the qualifications to receive this benefit^1^ (Klerman et al., 2012). Additionally, many eligible individuals choose not to take leave, or to return early, because they cannot afford to take extensive unpaid time off (Klerman et al., 2012).

Despite not having a widespread, paid parental leave policy, US states have the freedom to implement their own paid leave policies. However, as of 2016, the last year included in this study, only three states (California, New Jersey, and Rhode Island) had such laws in place (Brainerd, 2017). Private businesses in any state can also offer paid leave benefits if they choose to, but in 2017, only 16% of employees had access to paid benefits, and most of these employees were in higher status positions (Donovan, 2019; Isaacs et al., 2017).

Childcare in the US is primarily privately run and tends to be very expensive for children under 3 (OECD, 2020). Children older than three can attend preschool, but it is not always affordable or is only offered for half of the day (NCES 2020). When children reach age five in the US, they are old enough to begin kindergarten, which is federally funded, but in many states, kindergartens, like most preschools, are only held for half of the day (NCES, 2020).

Within the last couple years, there have been more rapid policy changes in the US at both the state and federal level. Three new states, New York, Washington, and Massachusetts, as well as Washington D.C., have enacted paid family leave policies that became effective as of 2020 (Bipartisan Policy Center, 2019). The entitlements offered by each state include gender-neutral parental leave with length ranging from four weeks to 12 weeks and benefits ranging from 55% of income to 90% (Bipartisan Policy Center, 2019). In addition to state-level policies, the Federal Employee Paid Leave Act (FEPLA) was signed into law in December of 2019 (AFGE, 2021). The law gives federal employees up to 12 weeks of paid time off after the birth or adoption of a new child and went into effect in October of 2020 (AFGE, 2021). Another bill introduced in December of 2019 allows working parents to collect a portion of their child-tax early to support them if they decide to take time off (Congressional Research Service, 2019). Even more recently, President Biden announced his American Families Plan which, if implemented, would drastically reform the current work-family system (The White House, 2021). It would provide support to low and middle income families by providing improved access to quality childcare, offer a comprehensive family and medical leave program, and extend tax credits for low and middle income families (The White House, 2021). These more recent policy developments in the US, while noteworthy, are not applicable to this study since the analysis ends in 2016. Future research should continue monitoring women’s employment outcomes in the aftermath of these policy changes.

## Methods

### Data

This study uses both individual and household-level data from the cross-national and repeated cross-sectional Luxembourg Income Study (LIS)—years 2000, 2004, 2010, and 2016—to analyze how gendered occupational segregation among parents has changed over time in Germany and the USA and how these patterns may differ for immigrants and native-born residents. The LIS database includes data related to income, wealth, employment, and demographic information for 53 industrialized countries. There have been over eleven waves of data collection spanning the years 1980–2018, though data from every country were not collected every year. The LIS staff collects the data, renders it comparable between countries, and makes it accessible to researchers worldwide. Scholars can access the data using the data center’s remote statistical interface.

Although sample size varies between countries and years, LIS data is robust and representative at the national level. For this analysis, sample sizes were restricted up front to include respondents who are (1) employed in the paid labor force, (2) the primary adults in each household, that is, the head of household or the spouse or cohabitating partner of the head of household, and (3) parents with their own children (ages 0–18) living in their home. With these restrictions applied, the sample of households and individuals in each year and each country—used in the regression estimation—are presented in Table 1. The unit of analysis in the regression models is at the individual level. Additionally, Table 1 includes descriptive statistics on the percentage of respondents not living with a partner, an important demographic group that later subsample analysis (included in the appendix) examines. Germany has a lower percentage of respondents not living with a partner at all time points except 2010, where the percentage jumps up about 10%, from 6.9 to 16.54%. Each observation provides full data for all explanatory variables included in the models, and there are no observations with missing estimation variables.

**Table 1:** Estimation sample sizes and percentage of respondents not living with a partner

	2000	2004	2010	2016
*Germany*				
Number of households	3513	2934	6141	5032
Number of observations	5133	4345	8647	7536
% Not living with partner	6.35%	6.90%	16.54%	11.01%
*USA*				
Number of households	31,668	29,491	26,142	22,612
Number of observations	49,246	45,185	39,409	34,417
% Not living with partner	11.58%	12.05%	12.14%	12.38%

### Measures

The primary dependent variable in the models is an occupational status measure. The variable comes from a question asking respondents to identify the classification of their first (or primary) job, and responses were re-coded by the LIS team according to ISCO-88 or ISCO-08 standards (ISCO, 2007). The ISCO-88 and ISCO-08 categorize occupational information based on the skill-level and skill-specialization required for the job. The occupational groupings created by ISCO systems are strongly correlated with occupational status, income, and job quality. I use a version of the measure that was collapsed (by the LIS team) from a ten-category occupation variable into a broader three-category variable where 1 = managers/professionals, 2 = other skilled workers, and 3 = laborer/elementary. The manager/professional category includes occupations that involve directing, leading, and creating policy or that require a high level of professional knowledge and skill (i.e. CEOs, scientists, doctors, lawyers, etc.). “Other skilled” occupations require technical knowledge, organizational skill, or skill in a craft or trade (i.e. technicians, associate professionals, clerical support workers, sales workers, crafts and trades professionals, etc.). “Elementary” occupations require knowledge and skill to perform simple and routine tasks (i.e. cleaners, agricultural workers, miners, food preparers, refuse workers, etc.).

A secondary dependent variable, used in solely in initial cross-tabulations, is respondent employment status. While I focus the bulk of the analysis on parents who *are* employed, I ran basic cross-tabulations of employment to provide greater context around the labor market situation in these two countries. Understanding the overall picture of who works in paid labor and who does not allows for clearer interpretation of my primary findings, which center on the occupational status of employed individuals. For example, if subsequent analysis were to show that employed mothers have similar levels of occupational prestige as fathers, it would be important to acknowledge that if they also have much lower employment rates, there is still inequality in the labor market. The employment variable is dichotomous: 1 = employed and 0 = unemployed. This variable was re-coded by the LIS team from a “labor force status” variable that identifies respondents’ self-assessed employment status. The variable originally distinguished between those who are employed, unemployed, and not in the labor force, but the transformed indicator variable collapses the unemployed and not in the labor force categories.

The primary independent variables in this analysis are the parent sex and immigrant variables. Respondent sex is a dichotomous indicator variable re-coded as 1 = fathers and 0 = mothers, since only parents are included in the analysis. Respondent immigrant status is also a dichotomous indicator, where 1 = immigrant and 0 = non-immigrant. Respondents are flagged as immigrants if (1) the data provider defined them as immigrant, (2) they self-define as immigrants, (3) they are citizens of another country, or (4) they were born in another country.

Control variables were theoretically motivated by existing literature and include indicators measuring family structure (Eckstein et al., 2019), self-reported health (Laaksonen et al., 2005), age (Ganzeboom et al., 1992), place of residence (Smith & Glauber, 2013), and socioeconomic status (Ganzeboom et al., 1992). Unfortunately, I could not include more demographically specific variables (i.e., fertility preferences, reasons for migrating, country of origin, etc.) that other authors writing on this topic have, since they are not included in the LIS data or were highly colinear with other variables in the models. In any case, the variables I have selected should be sufficient for the current analysis. The family structure variables are (1) marital status (1 = married, 2 = never married, and 3 = divorced/separated/widowed) where “married” serves as the reference group, living arrangements (1 = living with partner, 0 = not living with partner), and (2) children under four (1 = has own children under age four living in house, 0 = has own children age four to age 18 living in house). Since many of the family work policies implemented in Germany are directed at parents with children too young for school, the “children under four” variable captures targeted parents and allows for occupational status comparisons between parents with young children at home and parents with school-age children at home. Socioeconomic status is measured by level of education (eight categories ranging from “less than primary” to “doctorate or equivalent”, used in the model as a continuous variable), and household income (total income/1000). Other control variables include age, disabled (1 = disabled, 0 = not disabled), self-reported health (1 = good health, 0 = poor health), rurality (1 = rural, 0 = non-rural) and region (for Germany: 1 = East Germany, 0 = West Germany; for the US: 1 = Midwest, 2 = South, 3 = West, 0 = Northeast). In all models, the mean-centered versions of age and education are used, a practice that can alleviate “micro” multicollinearity and ensure that variance inflation factors (VIFs) remain low (Iacobucci et al., 2016).

### Analytic Strategy

To gain a clearer picture of overall employment rates for fathers and mothers at each time point, I ran cross-tabulations of the parent sex, immigration status, and employment variables at each time point. I also examined occupational status descriptively for immigrant and native-born fathers and mothers in both countries. Then, using used multinomial logistic regression with probability weighted data (using person weights provided by LIS) and clustered standard errors (by household), I predicted the likelihood of fathers’ and immigrants’ employment in “other skilled” and “labor/elementary” occupations *over* professional occupations and compared to mothers and non-immigrants. Multinomial regression is the best choice of model since I want to predict occupational status, a categorical variable, with a set of variables that are not all continuous (the control variables mentioned above). Rather than using simple logistic regression to analyze a binary occupational status outcome, I felt that the distinction between the three categories of occupational status were significant enough that combining, for example, the “other skilled” and “elementary” categories would not capture key ontological differences between these groups.

For the baseline specifications, I ran regression models with parent sex as the sole predictor variable and occupational status as the outcome variable as well as models with immigrant status as the sole predictor of occupational status. The intention here was to demonstrate the overall relationship the variables had with occupational status sans controls, to better understand how the control variables might affect the eventual significance of the main variables of interest. I then ran the primary models which included both the immigrant and parent sex variables along with all control variables. Finally, I computed the marginal effects for each variable in the model and recorded the point estimates of the effects, along with their p-values, in Table 3. In addition to running this analysis on the total sample, I also computed logistic regression models and marginal effects for two important sub-sample groups, mothers/fathers living with a partner or spouse and mothers/fathers living without a partner. Tables with these results are included in the appendix.

To answer my third research question, I then ran each of the primary models again with an interaction between parent sex and immigrant status. The interaction term allowed for comparisons between immigrant mothers, immigrant fathers, native-born mothers, and native-born fathers, an important distinction the models need to make if the literature is correct in concluding that the combination of being an immigrant and a mother can exacerbate disadvantage. In accordance with *American Sociological Review (ASR)* guidelines, I did not consider the statistical significance of the interaction coefficient in the models; rather, I computed the marginal predictions of the interaction and ran a Wald test to determine the equality of the effects (Mize, 2019; Mustillo et al., 2018). Finally, I plotted the predicted values of the interaction term to allow for easier comparisons between each demographic group in question.

## Results

### Descriptive Results

**Fig. 1:**
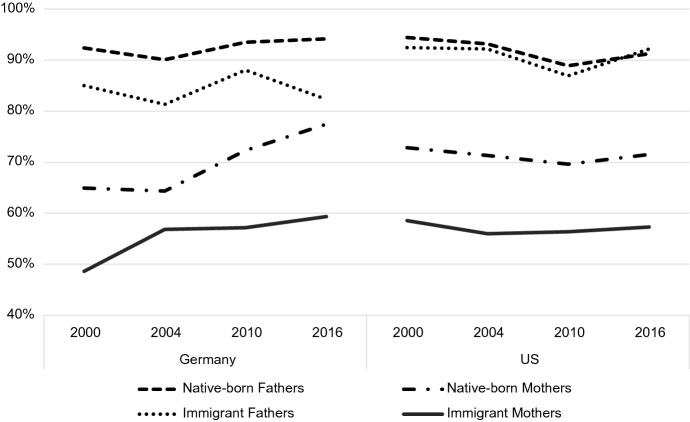
Mothers' and Fathers' employment rates by immigration status, country, and year

Figure 1 shows the descriptive employment rates for native-born and immigrant mothers and fathers in Germany and the US over the period of the study. While understanding employment in these two countries does not directly answer any of my research questions, it is necessary to understand the gendered context of the labor force in both countries for proper interpretation of the occupational status results. Rates for all subgroups in the US are relatively stable over time. US-born mothers’ rates hover around 70% in the USA, while immigrant mothers rates fall between 55 and 60%. Immigrant and native-born fathers in the US have quite similar rates both fluctuating around 90% over the period. In Germany, the story is similar, with immigrant mothers having the lowest rates (~ 50–60%), native-born and immigrant fathers having the highest rates (~ 80–95%), and native-born mothers falling in between (~ 65–80%). The most striking difference between the two countries is the upward trajectory of mothers’ employment (both native-born and immigrant) in Germany over time. Native-born mothers’ rates increase in the years following Germany’s work-related policy changes, but immigrant mothers began an upward trajectory in employment before policies were implemented, in 2004.

Both native-born and immigrant mothers are less likely than fathers to be employed, which indicates that the mothers included in the estimation sample below (restricted to employed respondents) represent a smaller fraction of the total population of mothers than the fraction of fathers in the estimation sample. Restricting the sample to employed parents might then introduce a selection effect, where the smaller fraction of mothers that opt into employment are qualitatively different than the larger percentage of fathers (perhaps more career-driven or successful) and the sample would then not reflect the labor market reality for all parents. To address this possible selection bias, I restrict the “target population” of the study to employed parents and emphasize that my findings and their implications only apply to the target population.

Figure 1 shows *what* gendered labor participation in Germany and the US looks like—in terms of raw employment rates—but it does not say much about *how* mothers and fathers participate in the labor market. The question of *how* parents’ experiences in paid employment differ can be explained in part by examining occupational status. The primary subject of this paper is to parse out whether the probability of attaining a certain occupational status changes over time depending on parent sex, immigrant status, or country context. As such, the rest of the analysis in this section focuses on changes in *occupation* over time, rather than changes in *employment* over time. To better address primary research questions, I exclude individuals who are not formally employed from the rest of the analysis.

Table 2 shows descriptively that in Germany, the percentage of parents in all immigrant and gender subgroups employed in professional occupations increases from 2000 to 2016 (with a dip in 2004), indicating that more professional jobs may have become available over the period. There are also consistent gaps in professional employment percentages between mothers and fathers and between immigrants and native-born individuals. In the US, the proportion of fathers and mothers in each occupational group have remained somewhat stable, relatively gender equal, and notably higher across the board than German proportions. There are more native-born mothers in professional positions than any other subgroup, including native-born fathers, at every time point, which is surprising given the extensive literature on the motherhood penalty in the US. In both countries, a much higher proportion of immigrants is employed in labor/elementary work and a particularly high proportion of immigrant mothers are employed in labor occupations in Germany.

**Table 2:** Percentage of immigrant and native-born mothers and fathers working in various occupational categories

	Germany				US			
	Immigrant		Native-born		Immigrant		Native-born	
	Mothers	Fathers	Mothers	Fathers	Mothers	Fathers	Mothers	Fathers
*2000*								
Manager/professional	8.07	12.51	17.81	28.66	24.05	28.65	36.74	36.46
Other skilled workers	67.55	76.08	74.42	67.01	60.81	56.79	57.22	57.57
Laborer/elementary	24.38	11.41	7.77	4.33	14.14	14.57	6.03	5.96
Total	100	100	100	100	100	100	100	100
*2004*								
Manager/professional	5.47	8.79	14.98	26.36	26.62	29.05	38.33	36.71
Other skilled workers	69.57	80.2	77.25	69.23	59.57	59.97	57.78	57.98
Laborer/elementary	24.96	11.02	7.77	4.4	13.81	10.98	3.89	5.31
Total	100	100	100	100	100	100	100	100
*2010*								
Manager/professional	15.03	16.51	21.55	33.02	27.41	25.59	38.29	35.52
Other skilled workers	67.2	75.15	72.03	63.33	58.46	58.49	58.75	57.40
Laborer/elementary	17.77	8.34	6.41	3.65	14.13	15.92	2.96	7.08
Total	100	100	100	100	100	100	100	100
*2016*								
Manager/professional	17.39	24.84	23.16	35.22	30.63	31.09	41.60	37.73
Other skilled workers	60.37	65.45	70.78	61.19	54.64	54.04	55.29	55.28
Laborer/elementary	22.24	9.7	6.07	3.59	14.73	14.87	3.11	7.00
Total	100	100	100	100	100	100	100	100

*Note* Descriptive statistics generated with weighted sample

### Main Effects

Baseline logistic models showed that immigrant status and parent sex were highly significant predictors of occupational status in both Germany and the US. While parent sex baseline models in several of the years in the US (2000, 2004, and 2016) initially returned insignificant results, parent sex became highly significant when the marital status variable was added to the model, indicating that the martial status and sex variables were likely associated. When the parent sex variable is no longer capturing the partial effect of marital status, it’s true and significant effect is revealed.

Subsequent models including all control variables and the model’s marginal effects (recorded in Table 3) show that in Germany, fathers are more likely than mothers to hold jobs in the professional/managerial sector across time. In the US, the situation is much different. US mothers are *more* likely than fathers to hold top tier occupations, as descriptive statistics suggested. Gaps between mothers and fathers in professional work are smaller at all time points in the US than they are in Germany, but US gaps are still statistically significant. In the “other skilled” occupational category, differences are much smaller and mostly non-significant in both countries. The marginal effects of labor jobs generally show the reverse pattern of professional jobs; German mothers are *more* likely to be employed in labor jobs than fathers and US mothers are *less* likely than fathers to be in labor jobs.

**Table 3:** Average marginal effects for regression model predicting occupational status

	2000		2004		2010		2016	
	Germany	US	Germany	US	Germany	US	Germany	US
Parent sex								
Female (ref.)								
Male								
Professional	0.063***	− 0.025***	0.067***	− 0.025***	0.050***	− 0.027***	0.062***	− 0.019***
Other skilled	− 0.021	0.026***	− 0.007	0.020***	− 0.006	− 0.008	− 0.018	− 0.009
Elementary	− 0.043***	− 0.001	− 0.059***	0.005	− 0.044***	0.035***	− 0.044***	0.027***
Immigrant status								
Native-born (ref.)								
Immigrant								
Professional	− 0.092***	− 0.057***	− 0.097***	− 0.040***	− 0.045*	− 0.052***	− 0.012	− 0.041***
Other skilled	0.032	0.015*	0.027	0.004	0.003	0.003	− 0.054*	− 0.007
Elementary	0.060***	0.042***	0.069***	0.036***	0.042***	0.049***	0.066***	0.048***
Marital status								
Married (ref.)								
Never married								
Professional	0.034	− 0.045***	− 0.015	− 0.082***	0.049	− 0.072***	0.042	− 0.061***
Other skilled	− 0.059	0.019	− 0.005	0.073***	− 0.028	0.045***	− 0.052	0.034**
Elementary	0.025	0.026***	0.020	0.008	− 0.021*	0.027***	0.010	0.027***
Divorced/separated/widow								
Professional	0.058*	− 0.017	0.007	− 0.040**	0.019	− 0.028*	− 0.007	− 0.020
Other skilled	− 0.084**	0.002	− 0.029	0.045***	− 0.033	0.007	0.004	0.003
Elementary	0.026	0.016*	0.022	− 0.005	0.015	0.021**	0.003	0.017*
Living arrangement								
Not living with partner (ref.)								
Living with partner								
Professional	− 0.032	− 0.013	− 0.031	− 0.045***	0.015	− 0.040**	− 0.053	− 0.010
Other skilled	− 0.020	− 0.013	− 0.034	0.037**	− 0.044	0.021	0.024	− 0.003
Elementary	0.052**	0.026***	0.065***	0.008	0.029*	0.019***	0.029*	0.013*
Children age								
Children ages 4–18 (ref.)								
Children under 4								
Professional	0.038*	0.007	− 0.027	0.020**	0.026	0.017**	0.029	0.008
Other skilled	− 0.013	− 0.011	0.036	− 0.016*	− 0.030	− 0.016*	− 0.011	− 0.001
Elementary	− 0.025*	0.003	− 0.008	− 0.004	0.004	0.000	− 0.018	− 0.007
Years of education								
Professional	0.061***	0.073***	0.052***	0.070***	0.064***	0.075***	0.063***	0.074***
Other skilled	− 0.039***	− 0.059***	− 0.040***	− 0.060***	− 0.047***	− 0.061***	− 0.050***	− 0.058***
Elementary	− 0.022***	− 0.014***	− 0.012***	− 0.010***	− 0.017***	− 0.014***	− 0.013***	− 0.016***
Age								
Professional	0.003**	0.002***	− 0.003*	0.002***	0.001	0.002***	− 0.001	0.002***
Other skilled	− 0.003**	− 0.002***	− 0.001	− 0.003***	− 0.002	− 0.002***	− 0.001	− 0.002***
Elementary	0.001	0.000*	0.003**	0.001**	0.001*	0.000	0.001*	0.000
Region								
West Germany/Northeast US (ref.)								
East Germany/Midwest US								
Professional	− 0.019	0.003	0.000	− 0.003	0.026	− 0.009	− 0.005	0.015
Other skilled	0.020	− 0.004	0.040*	0.002	− 0.020	0.004	0.024	− 0.012
Elementary	− 0.001	0.001	− 0.041**	0.001	− 0.006	0.005	− 0.020	− 0.003
South US								
Professional	–	0.017**	–	0.006	–	− 0.005	–	0.011
Other skilled	–	− 0.008	–	− 0.001	–	0.011	–	− 0.011
Elementary	–	− 0.009*	–	− 0.005	–	− 0.006	–	0.000
West US								
Professional	–	0.012	–	0.005	–	0.006	–	0.017*
Other skilled	–	− 0.015*	–	0.003	–	0.004	–	− 0.022*
Elementary	–	0.003	–	− 0.009*	–	− 0.010*	–	0.005
Rurality								
Suburban/urban (ref.)								
Rural								
Professional	− 0.003	− 0.021***	− 0.019	− 0.025***	− 0.013	− 0.004	− 0.038**	− 0.015*
Other skilled	− 0.014	0.004	0.020	0.024***	0.006	0.003	0.023	0.017*
Elementary	0.017	0.017***	0.000	0.001	0.007	0.001	0.015	− 0.002
Household income								
Professional	0.001***	0.001***	0.002***	0.001***	0.001***	0.001***	0.001***	0.000***
Other skilled	0.000	0.000	0.001*	0.000	0.001	0.000	0.000	0.000
Elementary	− 0.001	− 0.001***	− 0.003***	− 0.001***	− 0.002***	− 0.001***	− 0.002***	0.000***
Disability								
Non-disabled (ref.)								
Disabled								
Professional	0.038	− 0.076***	− 0.018	− 0.053**	− 0.098***	− 0.033	− 0.051	− 0.018
Other skilled	− 0.110*	0.060**	− 0.046	0.030	0.093**	0.029	0.024	0.034
Elementary	0.073*	0.016	0.064	0.023	0.005	0.003	0.027	− 0.016
Self-reported health								
Good/fair health (ref.)								
Poor health								
Professional	0.005	− 0.016	0.001	− 0.040	0.026	− 0.007	− 0.007	− 0.012
Other skilled	0.019	− 0.014	0.008	0.038	− 0.042	− 0.010	− 0.012	− 0.022
Elementary	− 0.025*	0.031	− 0.008	0.002	0.016	0.017	0.019	0.034

*p < 0.05, **p < 0.01, ***p < 0.001

Immigrants are less likely to be employed in professional occupations in both countries, but gaps are smaller for both countries in 2016 than they were in 2000 (Table 3). The gap shrinks particularly quickly in Germany. It was quite large (around 9%) in 2000 and essentially disappears by 2016. Again, the gaps in “other skilled” jobs are very small and largely not significant between immigrants and native-born individuals, indicating that most of the divergent outcomes are occurring at the extremes of the occupational spectrum. Finally, immigrants have a higher probability of working in labor occupations than native-born individuals across time and in both Germany and the US.

Control variables that have the most consistent association with occupational status include education, income, and age (Table 3). This is not surprising given that education, income, and occupational status are often used to represent the single concept of SES and must therefore be highly correlated. Age can often confound occupational outcomes, since older employed individuals may be further along in their careers and thus have higher occupational statuses (Ganzeboom et al., 1992). Marital status is also a consistent predictor of occupational status over time in the US, but not in Germany. Respondent living arrangements are associated with occupational status as well; in both Germany and the US, individuals living with their partner are more likely to be in labor/elementary positions, though the size of this effect shrinks over time. In the US, living in a rural community correlates with a lower probability of working in professional jobs in 2000, 2004, and 2016, a higher probability of working in either “other skilled” or labor jobs in 2000, 2004, and 2016. In Germany, rurality predicts lower occupational status only in 2016. Disabled individuals in Germany and the US tend to be less likely to work in professional jobs, but the effect disappears for both countries in 2016. Other variables that are sporadically and/or only marginally significant in both countries are having children under four, region, and self-reported health.

### The Combined Influence of Gender and Immigrant Status

**Fig. 2:**
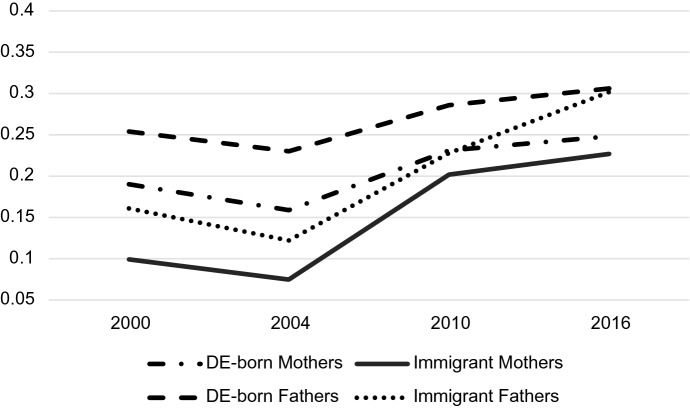
Predicted Probability of Employment in Professional/Managerial Occupations (Germany)

**Fig. 3:**
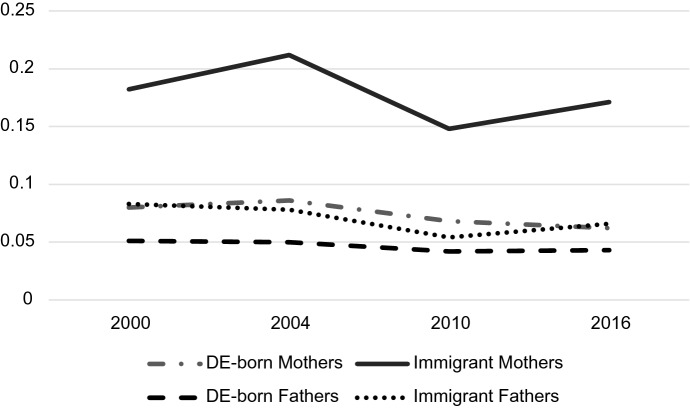
Predicted probability of employment in labor/elementary occupations (Germany)

**Fig. 4:**
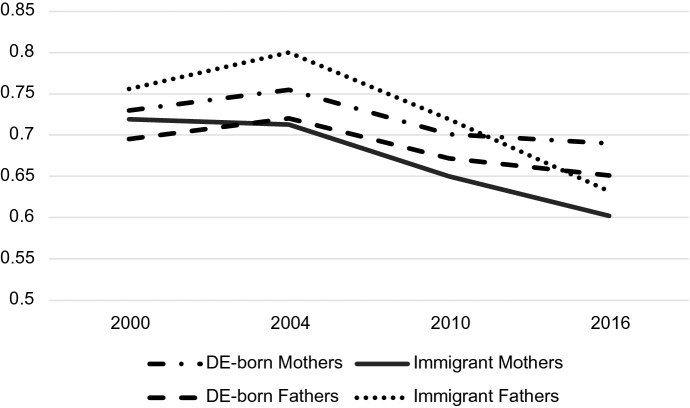
Predicted probability of employment in other skilled occupations (Germany)

**Fig. 5:**
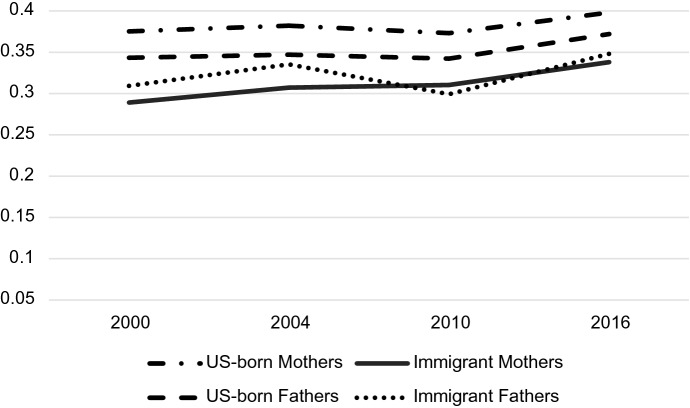
Predicted probability of employment in professional/managerial occupations (US)

**Fig. 6:**
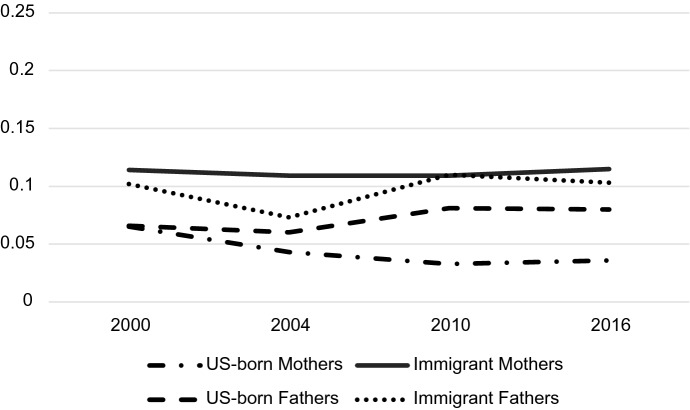
Predicted probability of employment in labor/elementary occupations (US)

**Fig. 7:**
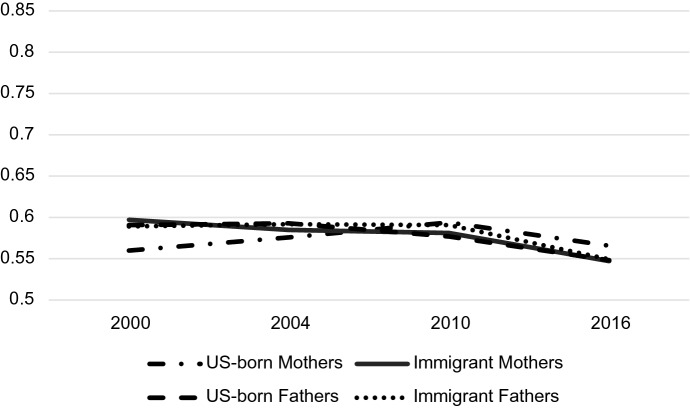
Predicted probability of employment in other skilled occupations (US)

Both parent sex and immigrant status mattered for predicting probabilities associated with occupation but understanding how they play together requires analyzing their interaction. Tables 4, 5 and 6 display the predicted probabilities, confidence intervals, and outcomes of a post-estimation Wald test for the parent sex-immigrant interaction. The predicted probabilities from this analysis are displayed graphically in Figs. 2, 3, 4, 5, 6 and 7. Notably, all interactions were significant in the US, but in Germany interactions in 2000 and 2004 were not significant, potentially due to smaller counts of immigrants in these years which contribute to greater standard errors and wider confidence intervals. However, the interaction was significant in 2010 and marginally significant in 2016 in Germany (Tables 4, 5, 6).

**Table 4:** Predicted probabilities of employment in managerial/professional occupations by gender and immigrant status in the US and Germany

	2000			2004				2010			2016		
	Predicted value	95% Conf. interval			Predicted value	95% Conf. interval		Predicted value	95% Conf. interval		Predicted value	95% Conf. interval	
*US predicted probabilities: managers/professionals*													
US-born Mothers	0.375	0.368	0.382	0.382		0.375	0.389	0.373	0.366	0.380	0.398	0.390	0.406
Immigrant Mothers	0.289	0.273	0.306	0.307		0.290	0.324	0.310	0.294	0.325	0.338	0.322	0.354
US-born Fathers	0.343	0.336	0.349	0.347		0.340	0.354	0.342	0.335	0.349	0.372	0.365	0.380
Immigrant Fathers	0.309	0.294	0.324	0.335		0.319	0.351	0.299	0.285	0.313	0.348	0.333	0.363
Wald test													
*Chi*-square stat	6.10			22.77				27.18			25.83		
*P*-value	0.0023			0.0000				0.0000			0.0000		
*Germany predicted probabilities: managers/professionals*													
DE-born Mothers	0.190	0.174	0.207	0.159		0.142	0.177	0.231	0.213	0.248	0.248	0.230	0.266
Immigrant Mothers	0.099	0.048	0.149	0.075		0.042	0.109	0.202	0.147	0.256	0.227	0.192	0.263
DE-born Fathers	0.254	0.238	0.270	0.230		0.212	0.248	0.286	0.266	0.306	0.306	0.284	0.329
Immigrant Fathers	0.161	0.121	0.200	0.122		0.087	0.158	0.228	0.188	0.267	0.302	0.250	0.353
Wald Test													
* Chi*-square stat	1.69			1.39				3.60			2.92		
* P*-value	0.1851			0.2485				0.0274			0.0538

**Table 5:** Predicted probabilities of employment in other skilled occupations by gender and immigrant status in the US and Germany

	2000			2004			2010			2016		
	Predicted value	95% Conf. interval		Predicted value	95% Conf. interval		Predicted value	95% Conf. interval		Predicted value	95% Conf. interval	
*US predicted probabilities: other skilled*												
US-born Mothers	0.560	0.553	0.568	0.576	0.568	0.583	0.594	0.586	0.602	0.566	0.557	0.574
Immigrant Mothers	0.597	0.579	0.616	0.585	0.566	0.604	0.581	0.563	0.599	0.547	0.529	0.565
US-born Fathers	0.591	0.584	0.599	0.593	0.585	0.600	0.577	0.569	0.585	0.548	0.538	0.557
Immigrant Fathers	0.589	0.572	0.605	0.592	0.575	0.609	0.591	0.575	0.607	0.549	0.532	0.566
Wald test												
*Chi*-square stat	6.10			22.77			27.18			25.83		
*P*-value	0.0023			0.0000			0.0000			0.0000		
*Germany predicted probabilities: other skilled*												
DE-born Mothers	0.730	0.708	0.752	0.755	0.728	0.781	0.701	0.680	0.722	0.690	0.669	0.712
Immigrant Mothers	0.719	0.658	0.781	0.713	0.649	0.777	0.650	0.588	0.712	0.602	0.553	0.650
DE-born Fathers	0.695	0.675	0.714	0.720	0.697	0.742	0.672	0.650	0.693	0.651	0.626	0.676
Immigrant Fathers	0.756	0.710	0.803	0.800	0.754	0.846	0.719	0.676	0.761	0.632	0.578	0.687
Wald test												
*Chi*-square stat	1.69			1.39			3.60			2.92		
*P*-value	0.1851			0.2485			0.0274			0.0538

**Table 6:** Predicted probabilities of employment in labor/elementary occupations by gender and immigrant status in the USA and Germany

	2000			2004			2010			2016		
	Predicted value	95% Conf. interval		Predicted value	95% Conf. interval		Predicted value	95% Conf. interval		Predicted value	95% Conf. interval	
*US predicted probabilities: laborer/elementary*												
US-born Mothers	0.065	0.061	0.069	0.043	0.039	0.046	0.033	0.030	0.037	0.036	0.032	0.040
Immigrant Mothers	0.114	0.102	0.125	0.109	0.097	0.120	0.109	0.099	0.120	0.115	0.104	0.126
US-born Fathers	0.066	0.062	0.070	0.060	0.056	0.065	0.081	0.076	0.086	0.080	0.075	0.086
Immigrant Fathers	0.102	0.093	0.112	0.073	0.065	0.082	0.110	0.099	0.120	0.103	0.093	0.113
Wald test												
*Chi*-square stat	6.10			22.77			27.18			25.83		
*P*-value	0.0023			0.0000			0.0000			0.0000		
*Germany predicted probabilities: laborer/elementary*												
DE-born Mothers	0.080	0.065	0.095	0.086	0.066	0.106	0.068	0.056	0.081	0.062	0.049	0.075
Immigrant Mothers	0.182	0.135	0.230	0.212	0.153	0.271	0.148	0.112	0.185	0.171	0.132	0.210
DE-born Fathers	0.051	0.040	0.063	0.050	0.036	0.064	0.042	0.033	0.052	0.043	0.030	0.055
Immigrant Fathers	0.083	0.054	0.112	0.078	0.046	0.109	0.054	0.038	0.070	0.066	0.044	0.088
Wald test												
*Chi*-square stat	1.69			1.39			3.60			2.92		
*P*-value	0.1851			0.2485			0.0274			0.0538		

In Germany, there is clearly convergence in gender and immigrant gaps in professional/managerial jobs over time (Fig. 2). Predictably, German-born fathers are the most likely group to be employed in professional/managerial jobs across time, and immigrant mothers are the least likely group to be employed in these jobs. However, the gap between immigrant mothers and native-born fathers (and by default, gaps between these groups and immigrant fathers/German-born mothers) shrinks considerably over time, with the most drastic changes occurring in 2010, the first measured time point after the parental leave policy change in 2007. The gap remains small in 2016, the time point after the childcare policy changes in 2013 and leadership policy changes in 2015. Additionally, the gap between immigrant and German-born fathers in professional jobs—a significant gap in 2000 according to predicted probability confidence intervals—begins to close in 2010 and completely disappears, demonstrated by largely overlapping confidence intervals, in 2016. Immigrant and native-born mothers show a similar trajectory with significantly different probabilities in 2000 and 2004, and nearly complete convergence by 2010 and 2016. There is, however, still a significant difference between professional probabilities of all mothers and all fathers in 2016. The convergence of native-born and immigrant parents in professional occupations may be due to recent influxes of educated immigrants from Eastern Europe (Sprengholz et al., 2021). It appears that all groups experienced increases in their likelihood of employment in professional jobs from 2000 to 2016.

Figure 3 demonstrates that although more immigrant mothers are participating in higher status professions in Germany over time, they are consistently much more likely to be employed in labor/elementary occupations. At all time points, the predicted probability of immigrant mothers employed in labor/elementary work was around 10% higher than any other group. Unlike the shrinking gender and immigrant gaps apparent in managerial/professional positions, in Germany, gaps in labor/elementary occupations for immigrant mothers remain large over time.

There do not appear to be any large gaps in “other skilled” professions over the period according to Fig. 4. Confidence intervals of marginal probabilities indicate that the only significant differences in skilled work are between immigrant fathers and native-born fathers in 2010 (with immigrant fathers being more likely to participate in other skilled work) and between immigrant and native-born mothers in 2016 (with native-born mothers being more likely to be employed in other skilled work).

Graphs for the US tell a different story. First, almost all demographic groups in the US have higher probabilities of working in managerial/professional jobs than any of the demographic groups in Germany (Fig. 5). In fact, German-born fathers, the highest achieving group in Germany, are consistently less likely than immigrant parents in the USA, the lowest-achieving groups in the US, of being employed in the top jobs. Second, the gaps between the various demographic groups are also much smaller at all time points than gaps in Germany. Figure 5 shows a slight convergence between groups over time, though the convergence appears much less dramatic than the convergence in Germany. Immigrant men also seem to take a different trajectory in the US than those in Germany—while German immigrant men experience an increase in their professional employment probability over time, US immigrant men experience a decline. Finally, US-born fathers are *less likely* than US-born mothers to be employed in professional/managerial jobs at all time points.

Figure 6 shows US predicted probabilities for labor/elementary occupations. Several differences between the US labor graph and the German labor graph are apparent. First, rather than solely immigrant mothers being the most likely group to be in labor jobs, immigrant mothers *and* immigrant fathers, share the higher probability (apart from 2004 where immigrant mothers do have a higher probability). In the US, the differing probabilities may have more to do with immigrant status than they do with the combination of immigrant status and gender. Second, the difference between US immigrants’ probability of being in elementary work and the probabilities of US-born parents is much smaller than the difference between immigrant mothers and all other subgroups in Germany. Finally, the gap between US-born mothers and fathers grows over the period, with fathers becoming increasingly likely to be employed in labor jobs and mothers becoming less likely participate in these jobs.

Figure 7 demonstrates the lack of variation in “other skilled” occupation participation in the US. All subgroups are clustered between predicted probabilities of 55% and 62% in 2000, and all groups converge further over time to about 55% to 57% in 2016. It seems that there is much less variation in probabilities for other skilled work by gender and immigrant status than variation in professional and labor occupations, indicating that the biggest gender/immigrant differences occur at the occupational poles. This is true of both the US and Germany.

## Discussion and Conclusions

Using LIS data, I investigate gendered and immigrant occupational segregation among parents in Germany and the US from 2000 to 2016. I find that parent sex and immigrant status are predictive of occupational status in both countries and across time. Surprisingly, the relationship between gender and occupational status is contrasting in Germany and the US; German-born fathers are more likely than mothers to hold higher status jobs and less likely to hold lower status jobs, while the opposite is true in the US. Immigrants in both countries are more likely to be in lower status work across time. An interaction between parents’ immigrant status and gender demonstrates closing gaps between immigrant and native-born parents in professional jobs in Germany but no closing gap by gender, as well as a consistently high predicted probability for immigrant mothers in labor employment compared to all other groups in Germany. It also reveals smaller gaps by gender and immigration status in the US at most time points. These findings suggest that mothers in Germany, and particularly immigrant mothers, still face substantial barriers in achieving occupational parity with fathers, especially (and unexpectedly) in comparison with the US.

Research most strongly supports the occupational patterns observed in Germany, where fathers fare better than mothers and native-born parents fare better than immigrant parents in occupational achievement (Blau et al., 2013; Cohen, 2013; Golash-Boza, 2015; Hall et al., 2019; Pettit & Hook, 2009). While US findings support the literature on immigrant disadvantages in occupational outcomes (Golash-Boza, 2015; Hall et al., 2019), they do not reflect the expected *gendered* occupational pattern. My analysis shows that US-born mothers have *higher* occupational statuses than fathers, though the practical significance of this finding is small. Research on US economic gender inequality finds that women, and especially mothers, are at a wage and status disadvantage (Blau & Kahn, 2017; Cohen, 2013; Correll et al., 2007; England, 2010; Gangl & Ziefle, 2009; Jee et al., 2019)*.* In contrast, this study finds that employed mothers in the US may not fare poorly compared to employed US fathers, at least for native-born mothers and when occupations are broken into three broad status categories.

For the most part, these gendered and immigrant patterns in the US hold constant over the 16-year period, while in Germany, striking positive changes in predicated probabilities of employment in professional/managerial jobs occur. All groups become more likely to be in professional jobs over time, particularly in 2010 and 2016 indicating general growth in the professional sector. Notably, immigrant/native-born gaps in professional occupations shrink in Germany over time as well, suggesting increasing occupational equality between immigrants and native-born Germans. The increase in immigrant probability of professional employment is probably due to a decreasing proportion of immigrants in “other skilled” occupations. The period saw a decline in immigrant employment in skilled work as immigrant probabilities of employment in professional work increased, but little to no change in labor/elementary probabilities.

The significance of the interaction between the identity of “mother” and the identity of “immigrant” is not consistent in either country. The literature that suggests overlapping marginalized identities, like being a woman with children *and* an immigrant, can harm individuals economically (Barglowski & Pustulka, 2018; Gomberg-Munoz, 2017; Hagan, 1998; Villares-Varela, 2018), and while this seems to be true in some years and for some occupational categories, it may not hold true across the board. In Germany, immigrant mothers are consistently much more likely than other groups to be employed in the lowest status work (Fig. 3), which supports the theoretical literature, but by 2010, their probability of being employed in the highest status jobs is not significantly different from native-born mothers (Fig. 2). US findings show that immigrant mothers’ probability of being employed in professional work is not significantly different from immigrant fathers’ probability, indicating that immigrant status may be a more limiting identity than gender in the US (Fig. 5). Additionally, 2004 was the only year that immigrant mothers were more likely than other groups to be in labor jobs in the US (Fig. 6).

This study supports research finding that gender occupational segregation is less severe in the US than it is in Germany (Blau & Kahn, 2013). Gender gaps in both high and low-status jobs are larger in Germany (greater difference in gendered probabilities of employment in each status sector), indicating that Germany may have further to go to achieve gender occupational integration than the US does. This is surprising given Germany’s consistently high rankings in the annual Global Gender Gap Report published by the World Economic Forum (World Economic Forum, 2020) and their progressive family work policies. Though I cannot make any causal claims about the efficacy of family work policies for improving mother’s labor market outcomes, the lack of change in mother-father gaps in occupational probabilities over the period indicates that the policies alone were not enough to thwart status-related gender gaps between parents.

As is the case with all research, this study is limited in a few ways. First, limitations with available data prevented me from including a variable to account for exogenous shocks, like the Great Recession during 2007–2009. In quasi- or natural experiments, accounting for these kinds of shocks is important and not being able to do so detracted from the ability of my study to produce causal findings. Because I do not claim that the research is an experiment, and is instead largely exploratory, my discussion of potentially confounding variables, like the recession, in the literature review is sufficient, but future research should include exogenous shock variables in statistical models to better account for their effects. Another modeling-related limitation is the selection effect produced by artificially limiting the sample to employed individuals, which creates a endogeneity problem where those who have a higher likelihood of finding a job are those in employment. Rather than capturing broad occupational outcomes for all mothers and fathers, this study can only comment on the outcomes of those who were employed at the time(s) of survey distribution. Because my research questions center around policies directed at working parents, I decided to accept this limitation, but it is an important caveat to keep in mind when considering the results of the study.

The short time frame of the study may also limit findings. Potential changes in occupational outcomes in Germany due to policy will likely play out in the long term since recipients of policy changes may only see gains in their career trajectories unfold over time. Researchers should continue to monitor occupational status in Germany (and the US) to determine longer term effects. Continuing to examine the occupational statuses of parents who utilize their benefits, compared to those that do not, will also be crucial to understanding the effects of the policies themselves, rather than the more ambiguous country-context factors cited in this study. Finally, I was unable to include country of birth variables in the model specification due to collinearity issues. Future research should certainly examine how country of origin influences gendered occupational status outcomes.

Despite its limitations, this study provides crucial information that is especially relevant now, in the midst of the COVID-19 pandemic. The pandemic exacerbated disparities in gendered employment outcomes in many countries, with mothers taking the hardest hits (Alon et al., 2020). It is unclear whether the pandemic has reversed the gendered dynamics in occupational status in the US, where mothers were more likely than fathers to be in higher status occupations in 2016. As new data is collected and published in LIS and other like datasets, these questions should be examined. Understanding how COVID-19—as well as future disasters—may set women with children back in terms of occupational status will be an important long-term research focus.

This study also points out that progressive work-family policies may not have the power to reverse gender inequality quickly, and they may not be enough on their own. Germany may have more federal-level policies in place, but my study shows that simply living in a country with progressive work-family policies does not directly correlate with occupational equality, at least in the short term. In fact, living a country with more haphazard policies, like the US, is associated with smaller disparities by gender and makes it more likely for native-born mothers to achieve higher occupational statuses. Germany could consider other ways to promote more equitable distributions of occupational outcomes. Finally, the paper highlights that immigrant women tend to fare worse than (or at least on par with) immigrant men in terms of occupational status in both countries. As such, governmental and non-governmental programs should direct specific efforts to assist immigrant women in the labor market rather than having programs that assist women only *or* immigrants only. The appendix tables, which capture more detailed occupational status differences between parents who live together with partners and those that live without partners, also provide useful information to program directors or policy makers interested in promoting occupational equality across other subgroups.

This study investigates questions of occupational inequality among immigrant and native-born parents in countries with vastly different work-family policy approaches. It illuminates the combined influence of gender and immigrant status on parents’ occupational outcomes. I find that both gender and immigrant status matter for occupational position individually, and that together, they matter even more. Surprisingly, US native-born mothers fare better than native-born fathers, whereas in Germany, mothers fare worse across the board. Gaps between groups in the predicted probability of occupational status are also larger in Germany than in the US, particularly between migrant mothers and German-born fathers. Implementing progressive work-family and women-in-leadership policies then is not a foolproof way to eradicate gender inequality, particularly for the most vulnerable groups in society, at least not in the short term. Future research should continue to analyze women’s occupational outcomes in Germany as time goes on, particularly among women that have utilized work-family benefits.

## Appendix

See Tables

**Table 7:** Average marginal effects for regression model predicting occupational status (with subsample not living with partner)

	2000		2004		2010		2016	
	Germany	US	Germany	US	Germany	US	Germany	US
N	326	5,703	300	5,443	1,430	4,785	830	4,261
Parent sex								
Female (reference)								
Male								
Professional	0.058	− 0.061***	− 0.006	− 0.047**	0.004	− 0.042**	0.003	− 0.008
Other skilled	− 0.001	0.050**	0.068	0.031	0.044	− 0.018	− 0.016	− 0.044*
Elementary	− 0.057	0.012	− 0.061	0.016	− 0.048*	0.061***	0.013	0.052**
Immigrant status								
Native-born (ref.)								
Immigrant								
Professional	− 0.057	− 0.081***	− 0.013	− 0.016	− 0.032	− 0.042**	0.015	− 0.057**
Other skilled	− 0.081	0.033	− 0.084	− 0.038	0.000	− 0.010	− 0.094	− 0.019
Elementary	0.139	0.048**	0.097	0.054***	0.032	0.051***	0.079	0.076***
Children age								
Children ages 4–18								
Children under 4								
Professional	− 0.070	− 0.003	− 0.018	− 0.027	0.035	− 0.016	− 0.122*	− 0.008
Other skilled	0.044	− 0.005	− 0.052	0.047*	− 0.020	0.005	0.162*	0.001
Elementary	0.026	0.009	0.070	− 0.020	− 0.015	0.011	− 0.040	0.007
Years of education								
Professional	0.046***	0.065***	0.033***	0.064***	0.047***	0.065***	0.051***	0.067***
Other skilled	− 0.016	− 0.047***	− 0.005	− 0.046***	− 0.017**	− 0.049***	− 0.035***	− 0.048***
Elementary	− 0.030*	− 0.018***	− 0.028	− 0.018***	− 0.029***	− 0.016***	− 0.016*	− 0.019***
Age								
Professional	0.001	0.001	0.000	0.002*	0.001	0.001	0.000	0.002*
Other skilled	− 0.005	− 0.003***	− 0.006	− 0.003**	− 0.003	− 0.003**	− 0.004	− 0.002*
Elementary	0.004	0.002**	0.006	0.001*	0.003*	0.001**	0.004	0.000
Region								
West Germany/Northeast US (ref.)								
East Germany/Midwest US								
Professional	− 0.082	− 0.012	− 0.055	− 0.017	0.025	− 0.023	− 0.013	0.012
Other skilled	0.069	− 0.013	0.159**	0.012	0.007	0.019	0.064	− 0.029
Elementary	0.013	0.025	− 0.104**	0.005	− 0.032	0.003	− 0.051	0.017
South US								
Professional	–	− 0.011	–	0.003	–	− 0.021	–	0.024
Other skilled	–	0.009	–	0.001	–	0.035	–	− 0.058**
Elementary	–	0.002	–	− 0.004	–	− 0.013	–	0.033*
West US								
Professional	–	0.014	–	0.024	–	− 0.032	–	0.018
Other skilled	–	− 0.043*	–	− 0.017	–	0.036	–	− 0.051*
Elementary	–	0.029*	–	− 0.007	–	− 0.003	–	0.033*
Rurality								
Suburban/urban (ref.)								
Rural								
Professional	0.044	− 0.014	0.014	0.005	− 0.021	− 0.009	− 0.034	− 0.028
Other skilled	− 0.137*	− 0.005	− 0.062	− 0.010	0.000	0.028	− 0.059	0.031
Elementary	0.093*	0.020	0.048	0.005	0.021	− 0.019	0.093*	− 0.004
Household income								
Professional	0.005***	0.002***	0.002**	0.002***	0.003***	0.002***	0.003***	0.001**
Other skilled	0.000	− 0.001	0.005*	0.000	0.002	− 0.001	0.005**	0.000
Elementary	− 0.005*	− 0.001**	− 0.007**	− 0.002***	− 0.005***	− 0.001***	− 0.008***	− 0.001*
Disability								
Non-disabled (ref.)								
Disabled								
Professional	− 0.192***	− 0.085*	− 0.103***	− 0.005	− 0.016	− 0.013	− 0.134***	0.010
Other skilled	0.268***	0.072	0.222***	− 0.008	0.022	0.057	0.149*	0.002
Elementary	− 0.076	0.012	− 0.119***	0.013	− 0.006	− 0.044*	− 0.015	− 0.011
Self-reported health								
Good/fair health (ref.)								
Poor health								
Professional	− 0.018	0.025	0.021	− 0.122**	− 0.023	− 0.002	− 0.049	0.011
Other skilled	0.018	− 0.071	− 0.015	0.137**	− 0.004	− 0.007	0.007	− 0.048
Elementary	0.001	0.046	− 0.006	− 0.015	0.027	0.009	0.043	0.037

**p* < 0.05, ***p* < 0.01, ****p* < 0.001

^7^ and

**Table 8:** Average marginal effects for regression model predicting occupational status (with subsample living with partner)

	2000		2004		2010		2016	
	Germany	US	Germany	US	Germany	US	Germany	US
N	4811	45,543	4047	39,742	7317	34,624	6726	30,156
Parent sex								
Female (reference)								
Male								
Professional	0.062***	− 0.024***	0.069***	− 0.025***	0.050***	− 0.027***	0.066***	− 0.020***
Other skilled	− 0.019	0.026***	− 0.009	0.020***	− 0.007	− 0.006	− 0.023	− 0.005
Elementary	− 0.043***	− 0.002	− 0.060***	0.005*	− 0.043***	0.033***	− 0.043***	0.025***
Immigrant status								
Native-born (ref.)								
Immigrant								
Professional	− 0.096***	− 0.054***	− 0.105***	− 0.042***	− 0.049*	− 0.052***	− 0.015	− 0.036***
Other skilled	0.040	0.014	0.038	0.009	0.004	0.005	− 0.046*	− 0.005
Elementary	0.056**	0.040***	0.067***	0.033***	0.045***	0.046***	0.061***	0.041***
Children age								
Children ages 4–18								
Children under 4								
Professional	0.039*	0.009	− 0.029	0.026***	0.025	0.021**	0.035	0.010
Other skilled	− 0.011	− 0.011	0.039	− 0.023**	− 0.031	− 0.019**	− 0.017	− 0.001
Elementary	− 0.028*	0.002	− 0.010	− 0.003	0.006	− 0.002	− 0.018	− 0.009*
Years of education								
Professional	0.062***	0.074***	0.054***	0.071***	0.065***	0.077***	0.064***	0.076***
Other skilled	− 0.041***	− 0.060***	− 0.043***	− 0.062***	− 0.050***	− 0.063***	− 0.052***	− 0.060***
Elementary	− 0.021***	− 0.014***	− 0.011**	− 0.009***	− 0.016***	− 0.014***	− 0.012***	− 0.016***
Age								
Professional	0.002*	0.002***	− 0.003*	0.003***	0.000	0.002***	− 0.001	0.002***
Other skilled	− 0.003*	− 0.002***	0.000	− 0.003***	− 0.002	− 0.002***	0.000	− 0.002***
Elementary	0.000	0.000	0.003**	0.000*	0.002*	0.000	0.001	− 0.001*
Region								
West Germany/Northeast US (ref.)								
East Germany/Midwest US								
Professional	− 0.010	0.005	0.006	0.001	0.032	− 0.006	0.005	0.016
Other skilled	0.011	− 0.003	0.023	− 0.001	− 0.028	0.001	0.007	− 0.009
Elementary	− 0.001	− 0.002	− 0.029*	0.000	− 0.003	0.005	− 0.012	− 0.006
South US								
Professional	–	0.021**	–	0.009	–	− 0.001	–	0.011
Other skilled	–	− 0.009	–	− 0.003	–	0.007	–	− 0.004
Elementary	–	− 0.011*	–	− 0.006	–	− 0.006	–	− 0.007
West US								
Professional	–	0.012	–	0.003	–	0.011	–	0.017*
Other skilled	–	− 0.010	–	0.006	–	0.001	–	− 0.017
Elementary	–	− 0.001	–	− 0.009*	–	− 0.012*	–	0.000
Rurality								
Suburban/urban (ref.)								
Rural								
Professional	− 0.009	− 0.020***	− 0.022	− 0.028***	− 0.012	− 0.002	− 0.036*	− 0.012
Other skilled	− 0.003	0.005	0.028	0.028***	0.008	− 0.001	0.029	0.014
Elementary	0.011	0.015***	− 0.006	0.000	0.004	0.003	0.007	− 0.003
Household income								
Professional	0.001***	0.001***	0.002***	0.001***	0.001***	0.001***	0.001***	0.001***
Other skilled	0.000	0.000	0.001	0.000	0.000	0.000	0.000	0.000
Elementary	− 0.001	− 0.001***	− 0.002***	− 0.001***	− 0.001***	− 0.001***	− 0.002***	0.000***
Disability								
Non-disabled (ref.)								
Disabled								
Professional	0.046	− 0.072***	− 0.014	− 0.067***	− 0.106***	− 0.036	− 0.040	− 0.026
Other skilled	− 0.134**	0.055**	− 0.058	0.041	0.103**	0.016	0.009	0.043*
Elementary	0.089*	0.017	0.072	0.026	0.003	0.019	0.031	− 0.017
Self-reported health								
Good/fair health (ref.)								
Poor health								
Professional	0.015	− 0.031	− 0.001	− 0.006	0.033	− 0.008	0.000	− 0.016
Other skilled	0.015	0.003	0.008	0.000	− 0.052	− 0.009	− 0.016	− 0.018
Elementary	− 0.030**	0.029	− 0.007	0.005	0.018	0.017	0.016	0.034

**p* < 0.05, ***p* < 0.01, ****p* < 0.001

^8^.

## Footnotes

1 These qualifications include 1) working in a firm with at least 50 employees, 2) working for the firm for at least one year, and 3) having at least 1,250 h of service in the previous year (24 h a week).

## Declarations

### Availability of Data and Material

The datasets analyzed in this study are publicly available on the LIS Cross-National Data Center website: https://www.lisdatacenter.org/our-data/lis-database/.

### Code Availability

The code used to carry out this analysis can be made available by the author upon reasonable request.
